# Satisfaction Levels concerning Current Chronic Constipation Treatment Options in Saudi Arabia

**DOI:** 10.1155/2020/6545121

**Published:** 2020-01-02

**Authors:** Abdulaziz Alhossan, Ziyad Alrabiah, Sultan M. Alghadeer, Syed Wajid, Mohamed N. Al-Arifi, Salmeen D. Babelghaith

**Affiliations:** Department of Clinical Pharmacy, College of Pharmacy, King Saud University, Riyadh, Saudi Arabia

## Abstract

**Methods:**

This study was undertaken in Riyadh City, Saudi Arabia, in April 2019. The study population comprised respondents aged ≥18 years who had been recruited to participate through advertising on social media.

**Results:**

Of 532 respondents who completed our questionnaire, 153 (25.4%) had constipation, based on listed criteria, and of these, 121 (22.7%) reported having been constipated for ≥6 months; nearly 48% reported having been constipated for >3 years, while 63.6% of those chronically constipated were female. Bisacodyl was the laxative medication most used to treat chronic constipation, and 17.4% of users reported that they had used laxative medication for >12 months. Complementary and alternative medicines (CAMs) were utilized in 44.4% of respondents with chronic constipation. There was a significant association between the group who used CAMs and age (*p* = 0.013). Drinking water was the most common CAM adopted for those experiencing chronic constipation, followed by consumption of fiber (35.5%) and senna (19.8%).

## 1. Introduction

Constipation is a frequently occurring complaint that is considered to be underreported, as people with constipation have been reported to not generally seek medical advice [1]. Constipation is a functional disorder of the gastrointestinal tract, which can result in irregular stools and the difficult and painful passage of hard, solid stools. Severe constipation can result in digestive tract occlusion, and treatment may require a medical procedure [2].

Constipation has been defined as less than three bowel movements per week with one of the following signs: pain during defecation, solid feces, and a feeling of incomplete emptying [1, 2].

One study conducted in Central Saudi Arabia reported a prevalence rate for constipation of approximately 4.4% and that 83.3% of people in Riyadh City suffered from constipation [2]. Another study also conducted in Central Saudi Arabia was aimed at determining the incidence of constipation using three criteria, namely, self-perception, Rome III criteria, and Bristol criteria, for the diagnosis of constipation. That study found that the prevalence of constipation among the general population was 43%, 60%, and 25%, based on these three criteria, respectively [3]. However, this study did not report the prevalence rates concerning chronic constipation, which may present as a condition of long duration. A United States study reported chronic constipation in 55% of their study population, of whom 43% had experienced chronic constipation for ≥4 years [3].

Along with problematic side effects, health-related quality of life (QoL) has been reported to be adversely affected in individuals with chronic constipation [3–5], and one study showed that the level of serious side effects adversely correlated with a patient's apparent QoL [3]. Public health studies have reported that in patients with chronic constipation, a poor QoL was a key indicator for medical service use and resulting healthcare costs [3, 6]. Further studies have stated that patients who seek therapeutic treatment for chronic constipation (reported to be 25% among those with chronic constipation) are not always treated adequately and are frequently dissatisfied with treatment results [1, 3].

Treatment for chronic constipation can be challenging, and there are differing etiologies [1, 7]; therefore, treatment should address the fundamental pathophysiology while addressing the various manifestations related to this condition [1]. Currently, lifestyle and dietary modifications are advised prior to the utilization of laxatives in the treatment of chronic constipation [8]; however, limited information is available on the efficacy of these lifestyle and dietary modifications [9, 10]. If modifications to diet and lifestyle fail to have an effect, laxative use is recommended as the first-line pharmacological management [1, 10]. Laxatives are classified according to their method of activity and comprise bulking agents (e.g., fiber), stimulant laxatives (e.g., bisacodyl and sodium picosulfate), stool conditioners (e.g., docusate), and osmotic laxatives (e.g., polyethylene glycol and lactulose) [10]. Numerous studies have shown that laxative drugs increase the number of bowel motions, and some studies have reported improvements in side effects; however, these studies were limited and did not report clinically important endpoints [7]. One study undertaken in the United States that investigated a population with chronic constipation reported that 72% of that study population used laxatives and 47% reported dissatisfaction with their treatment [1].

To date, there have been very few studies published in the Middle East, particularly in Saudi Arabia, to assess treatment satisfaction among people suffering from chronic constipation. This study was undertaken to determine the prevalence rate concerning chronic constipation and to assess satisfaction with current treatment options among people with chronic constipation.

## 2. Methods

### 2.1. Study Design

We used an online questionnaire for respondents to answer questions concerning their satisfaction with current treatments and to determine the prevalence of chronic constipation and identify treatment options available to people suffering from chronic constipation. This study was undertaken in Riyadh City, in Central Saudi Arabia, in April 2019.

### 2.2. Study Population

The study population comprised respondents aged ≥18 years, who had been recruited via advertising on social media (e.g., Twitter and WhatsApp). The study inclusion criteria comprised a self-report in regard to chronic constipation, which was characterized as <3 bowel movements per week plus one or more of the following signs for at least 6 months: (1) pain during evacuation, (2) clumpy/solid feces, and (3) a feeling of incomplete evacuation.

### 2.3. Sample Size

According to a previously reported prevalence rate for constipation of 60% [3], the sample size was calculated using the following formula:

(1) $$\begin{array}{c} n=\frac{z^{2}\times p\times q}{d^{2}}, \end{array}$$

where *n* is the minimum sample size, *z* is the constant (1.96), *p* is the prevalence of constipation (0.6%), *q* = (1 − *p*), *Z* is the standard normal deviation of 1.96 which corresponded to a 95% confidence interval, and *d* is the desired degree of accuracy:

(2) $$\begin{array}{c} n=\frac{{\left(196\right)}^{2}\times 0.6.\times \left(1-0.6\right)}{{\left(0.05\right)}^{2}}, \end{array}$$

where *n* = 370 respondents.

### 2.4. Survey

A survey undertaken to obtain data for this study was validated by the authors. The survey was modified from a survey reported in a similar study [1]. The questionnaire comprised two sections. One section included population demographics such as age, sex, and educational levels. The second section sought information concerning constipation symptoms and details concerning any history of constipation, treatment, and satisfaction with treatment. We conducted a pilot study involving 13 individuals, and Cronbach's alpha validity for the survey was 0.83.

### 2.5. Data Analysis

We used the Statistical Package for the Social Sciences, version 25, to analyze the data. Descriptive statistics and chi-squared tests were also performed.

## 3. Results

Overall, 532 respondents completed the questionnaire, of whom 153 (25.4%) had constipation, based on the listed criteria. In total, 121 (22.7%) respondents had constipation for ≥6 months. Nearly 48% of respondents reported having constipation for >3 years, and 63.6% of the respondents with chronic constipation were female. Among those with chronic constipation, 50% were aged between 18 and 24 years and 66.9% of respondents with chronic constipation had been university educated (Table 1).

Table 2 is shown.

### 3.1. Constipation Management and Respondents' Satisfaction with Treatment

In total, 63.6% of respondents with chronic constipation did not use laxative medication. Bisacodyl was the laxative medication most used (Table 2).

This study only found relationship between the CAM users and duration of constipation (*p* = 0.019) as shown in Table 3.

Out of 44 respondents who used laxative medication, only 27.3% reported that they were either very satisfied or satisfied with their medication, 40.9% of the respondents were neutral, and 27.2% of the respondents were dissatisfied (Table 4). Respondents were asked if they were interested in considering other treatment options to manage their constipation, and they responded as follows: 43.2% stated they were very interested, 20.5% indicated they were probably interested, 18.2% did not know if they were interested, 11.4% indicated they were probably not interested, and 6.8% indicated no interest in other treatment options (Table 4).

### 3.2. The Use of Complementary and Alternative Medicines (CAMs) Used to Treat Chronic Constipation

This study found that 44.4% of respondents used CAMs to treat their chronic constipation. There was a significant association between CAM users and age (*p* = 0.013). Drinking water was reported as the most commonly used CAMs to treat chronic constipation, followed by fiber (35.5%) and senna (19.8%) (Tables 5 and 6).

## 4. Discussion

The prevalence rate for chronic constipation among respondents in our study was 22.7%. Their chronic constipation was frequently of long duration, with 47.9% of respondents reportedly having constipation for >3 years. While some studies have reported prevalence rates for general constipation among the Saudi population, no published studies have reported on prevalence rates for chronic constipation in Saudi Arabia; therefore, our findings cannot readily be compared to previous findings. However, our study findings are similar to a previous Europe-based study that reported a high percentage of the population who had experienced chronic constipation for >3 years [1]. The prevalence rates for constipation have been shown to increase in relation to age [11–13]. In addition, the largest percentage of participants, namely, those aged between 34 and 51 years, had chronic constipation. In a cross-sectional study by Wald et al., nearly 50% of participants aged between 30 and 59 years responding to a self-administered Rome III criteria questionnaire were found to have constipation [14]. Additionally, 63.6% of the participants with chronic constipation were females, and this finding is similar to two other studies conducted in Saudi Arabia [1, 15]. However, constipation in women is most commonly due to pregnancy and childbirth, which may be related to female hormonal changes associated with digestive system disruption [15].

Patients with chronic constipation are first advised to make dietary and lifestyle changes. If these recommendations fail, then conventional laxative drugs can be used [1, 16]. Laxative drug usage has been reported to be high among people experiencing chronic constipation [1]. In contrast, only 36.4% of the chronically constipated respondents in our study indicated that they had been using laxative drugs to treat their constipation, while a Europe-based study reported that 68% of study participants with chronic constipation used laxative drugs [1]. Another study undertaken in the United States showed that 72% of participants with constipation reported using laxatives drugs [3]. The laxative drugs most used by the respondents in that study were bisacodyl (14.9%) and polyethylene glycol (14%). To date, there have been no studies published concerning laxative medication use in Saudi Arabia; thus, more studies are needed to provide a comparison with our results. However, in the Europe-based study, polyethylene glycol was the most common laxative used by participants with chronic constipation [1].

In our study, 27.3% of respondents with chronic constipation were satisfied with their laxative medication, whereas in the study undertaken in the United States, 47% of those taking laxatives were dissatisfied with their medication [3]. The results from the Europe-based study showed that 28% of patients with chronic constipation were either satisfied or very satisfied with their laxative medication [1]. Our study did not investigate reasons for dissatisfaction with treatment; however, Johanson and Kralstein [4] concluded that the most common reasons for dissatisfaction were that the laxative medication did not appear to work well and that there were side effect and adverse effect issues [3]. Moreover, the researchers reported that while 146 patients used over-the-counter laxatives, 71% were not totally satisfied with the efficacy of the products, 60% stated that the laxatives failed to relieve many of their constipation symptoms, and 44% considered that the medication did not totally relieve their constipation [3].

With low satisfaction levels in regard to laxative medication, patients are likely to seek other approaches to treatment. In this survey, 44.4% of respondents with chronic constipation reported the use of CAMs. Most respondents with chronic constipation (66.9%) reported that drinking water was useful for chronic constipation, and 35.5% reported increasing their fiber intake to relieve their constipation. These results are similar to those of previous studies that considered that successful treatment for constipation should include lifestyle changes involving, for example, a more suitable diet containing fruit, fluids, and probiotics [17, 18].

## 5. Limitations

This study was the first to investigate treatment options identified by respondents suffering from chronic constipation in Saudi Arabia; however, there are some limitations. The study design involved an online-conducted self-report survey, which is likely to have introduced a degree of selection bias towards those with constipation symptoms and those who were able to respond to the questionnaire in April 2019 through social media, as well as presuming an accurate reporting and understanding of symptoms. The current population of Riyadh is estimated to be around 7,000,000 with almost 55% constituting the male and 45% constituting the female distribution in the city. Despite the fact that the sample size was calculated, the inherent nongeneralizability of online social media sampling cannot be excluded. However, the study focused on a general population and was not limited to patients who had consultations at the hospital. Most Saudi social media users are young people who were representing the age distribution in our study. Therefore, the findings of this study could only represent the situation among social media users in Riyadh City, but not to the whole Riyadh City residents.

## 6. Conclusions

Our results showed that 22.7% of the respondents reportedly had chronic constipation and that 63.6% of the respondents had not used laxative medications. Only 27.3% reported that they were satisfied with their medication. These results suggest a need for treatment alternatives for patients who remain dissatisfied with current treatment options as well as further research on why existing treatments appear to be less effective for some patients with chronic constipation.

## Figures and Tables

**Table 1:** Demographic data concerning the frequency of chronic constipation (*n* = 121).

Variables	*n*	%
Sex		
Male	44	36.4
Female	77	63.6
Age group (years)		
18-24	93	54.5
25-33	29	22.3
34-51	25	19.0
52-64	5	3.3
≥65	1	0.8
Employment status		
Student	65	53.7
Unemployed	21	17.4
Employed	35	28.9
Education		
None	2	1.7
Primary/secondary	4	3.3
High school	25	20.7
University	81	66.9
Postgraduate	9	7.4
Insurance		
None	52	43.0
Government hospital	48	43.0
Private	21	17.4
Duration of constipation		
6-12 months	26	21.5
1-2 years	26	21.5
2-3 years	11	9.1
>3 years	58	47.9
Use of complementary and alternative medicines (CAMs)		
Yes	48	39.7
No	73	60.3

^∗^Missing data.

**Table 2:** Type of laxative use and its duration.

	*n*	%
Laxatives use		
Yes	44	36.4
No	77	63.6
Laxatives type^∗^		
MiraLAX	4	3.3
Bisacodyl	17	14.0
Others	18	14.9
No use of laxatives	77	63.6

^∗^Missing data.

**Table 3:** Respondents' demographic data concerning the duration of constipation (*n* = 121).

Duration of constipation						
Variables	6-12 months, *n* (%)	1-2 years, *n* (%)	2-3 years, *n* (%)	>3 years, *n* (%)	Total, *n* (%)	*p* value^∗^
Sex						
Male	8 (18.2)	12 (27.3)	5 (11.4)	19 (43.2)	44 (36.3)	0.544
Female	18 (23.4)	14 (18.2)	6 (7.8)	39 (50.6)	77 (63.6)	
Age group						
18-24	16 (24.3)	18 (27.3)	6 (9.1)	26 (39.4)	66 (54.5)	
25-33	7 (25.9)	5 (18.5)	3 (11.1)	12 (44.4)	27 (22.3)	0.083
34-51	2 (8.7)	1 (4.3)	1 (4.3)	19 (82.6)	23 (19.0)	
52-64	1 (25.0)	1 (25.0)	1 (25.0)	1 (25.0)	4 (3.3)	
>65	—	1 (100)	—	—	1 (0.8)	
Laxatives^∗∗^						
MiraLAX	3 (75.0)	1 (25.0)	—	—	4 (3.3)	0.160
Bisacodyl	3 (17.6)	4 (23.5)	2 (11.8)	8 (47.1)	17 (14.0)	
Others	5 (27.8)	2 (11.1)	—	11 (61.1)	18 (14.9)	
Not using laxatives	13 (16.9)	18 (23.4)	9 (11.7)	37 (48.1)	77 (63.6)	
CAM use						
Yes	5 (7.4)	10 (14.7)	5 (7.4)	28 (41.2)	48 (39.7)	0.019
No	21 (24.7)	16 (18.8)	6 (7.1)	30 (35.8)	73 (60.3)	
Education						0.057
None	—	—	—	2 (100)	2 (1.6)	
Primary/secondary	2 (50.0)	—	2 (50.0)	—	4 (3.3)	
High school	5 (14.7)	8 (23.5)	2 (5.9)	10 (29.4)	25 (20.7)	
University	16 (15.8)	17 (16.8)	5 (5.0)	43 (42.6)	81 (66.7)	
Postgraduate	3 (25)	1 (8.3)	2 (16.7)	3 (25.0)	9 (7.4)	

^∗^Chi-squared test, ^∗∗^ missing data.

**Table 4:** Satisfaction with chronic constipation management (*n* = 44).

Variables	*n*	%
Are you satisfied with the treatment you are receiving for chronic constipation?^∗^		
Very satisfied	3	6.8
Satisfied	9	20.5
Neutral	18	40.9
Dissatisfied	10	22.7
Very dissatisfied	2	4.5
Are you interested in additional products for chronic constipation that could relieve your symptoms faster?		
Yes, absolutely	19	43.2
Probably	9	20.5
Do not know	8	18.2
Probably not	5	11.4
No, absolutely not	3	6.8

^∗^Missing data (*n* = 4).

**Table 5:** The association between the demographic characteristics of respondents with chronic constipation and CAM use.

Characteristics	CAM users				*p* value^∗^
	Yes, *n* %		No, *n* %		
Sex					
Male	17	38.6	27	61.4	1.0
Female	31	40.3	46	59.7	
Age group (years)					
18-24	22	33.3	44	66.7	0.013
25-33	8	29.6	19	70.4	
34-51	15	65.2	8	34.8	
52-64	3	75.0	1	25.0	
>64	—	—	1	100	
Educational level					
Illiterate	1	50.0	1	50.0	
Primary/secondary school	1	25.0	3	75.0	0.836
High school	8	32.0	17	68.0	
University	35	43.2	46	56.8	
Postgraduate	3	33.3	6	66.7	

^∗^Chi-squared test.

**Table 6:** Types of CAM used for chronic constipation management^∗^.

CAM type	*n*	%
Senna	24	19.8
Acupuncture	6	5.0
Massage	12	9.9
Castor oil	19	15.7
Fiber	43	35.5
Drinking water	81	66.9
Coffee	17	14.0
Others	4	3.3

^∗^Patient used more than one form of CAM.

## Data Availability

The data used to support the findings of this study are available from the corresponding author upon request.
